# Drivers of Soil Carbon Variability in North America’s Prairie Pothole Wetlands: A Review

**DOI:** 10.1007/s13157-025-01898-9

**Published:** 2025-01-30

**Authors:** Chantel J. Chizen, Angela K. Bedard-Haughn

**Affiliations:** 1 Department of Soil Science, College of Agriculture and Bioresources, University of Saskatchewan, Saskatoon, SK Canada; 2 Global Institute for Water Security, University of Saskatchewan, Saskatoon, SK Canada

**Keywords:** Prairie Pothole Region, Wetland soil carbon, Climate gradients, Water management, Agricultural wetlands

## Abstract

There is an ongoing demand for region-specific soil organic carbon estimates to support sustainable land management and inform carbon credit programs. The Prairie Pothole Region is prominent agricultural area that extends through Canada and the United States, and features a significant number of wetlands commonly referred to as prairie potholes. The contribution of these wetlands to landscape-level soil organic carbon storage is complex and may not be consistent across the region as influenced by several environmental and management factors. This study reviews existing literature to identify the main factors that contribute to variability in soil organic carbon stocks in prairie pothole wetlands. Soil organic carbon stock data from 10 studies in the Prairie Pothole Region were summarized through a meta-analysis. Variable importance and regression analyses were used to assess which factors explain variability in soil organic carbon. Wetland class explained up to 26.6% of the variability in soil organic carbon. Other important factors included ecoregion as well as land management. There were significant differences in average wetland soil organic carbon stocks across the ecoregions. Data limitations restricted our ability to accurately estimate the stocks for wetland class and land management. The findings from this study highlighted the need for targeted studies in the Northern short grassland ecoregion as well as studies that consider wetland classes under various land uses. To advance wetland carbon research in the Prairie Pothole Region, recommendations were provided on landscape-level carbon modelling, soil carbon measurement and monitoring, and improved wetland classification systems.

**Graphical Abstract:**

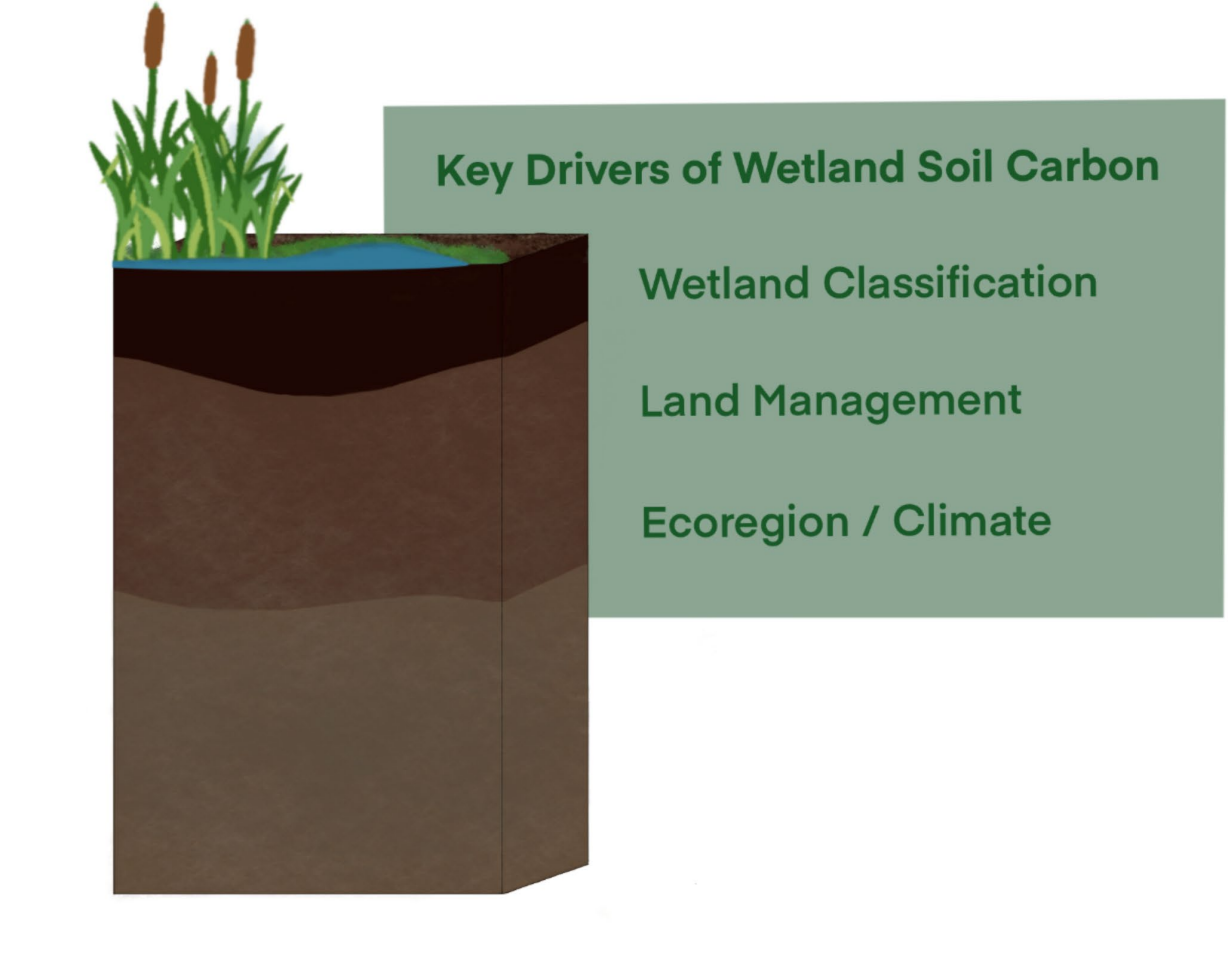

**Supplementary Information:**

The online version contains supplementary material available at 10.1007/s13157-025-01898-9.

## Introduction

### Wetlands in the Prairie Pothole Region

Wetlands are known for their ecosystem services, such as nutrient retention, drought and flood mitigation, water quality improvements, wildlife habitat, and carbon sequestration, which provide essential benefits to society. The overall value of ecosystem services for wetlands is generally greater than many other ecosystems (Mitsch et al. 2015; Millennium Ecosystem Assessment 2005). However, not all wetlands contribute the same services and the extent of these services can differ with wetland type, management, and time (Gleason et al. 2008; Watanabe and Ortega 2011; Pindilli 2021; Whitfield et al. 2024). The hydrological and biogeochemical cycles that occur in healthy, functioning wetlands are the main processes that enable the provisioning of these ecosystem services (Watanabe and Ortega 2011).

A notable regulating ecosystem service provided by wetlands is soil carbon sequestration, which is transfer of atmospheric carbon to the soil carbon pool. While carbon cycling inherently involves both gains and losses between the atmospheric and soil carbon pools, sequestration represents a net accumulation of soil organic carbon over time. Soil organic carbon sequestration directly promotes climate regulation but this carbon that is stored in the soil also supports other ecosystem services such as food provisioning and water storage by enhancing the soil fertility and water holding capacity (Pindilli 2021). Soils have a carbon storage capacity as influenced by factors including soil texture, land management, climate, and hydrology. Carbon stocks are measured or estimated values of soil organic carbon storage. These stock values provide a snapshot of the amount of carbon stored in the soil carbon pool at a given time, as well as under specific environment and/or management conditions. Like any other ecosystem service, soil organic carbon sequestration and storage capacity varies among wetlands. This variability highlights the need for regional data to accurately assess the contributions of wetlands to soil organic across diverse landscapes.

**Fig. 1:**
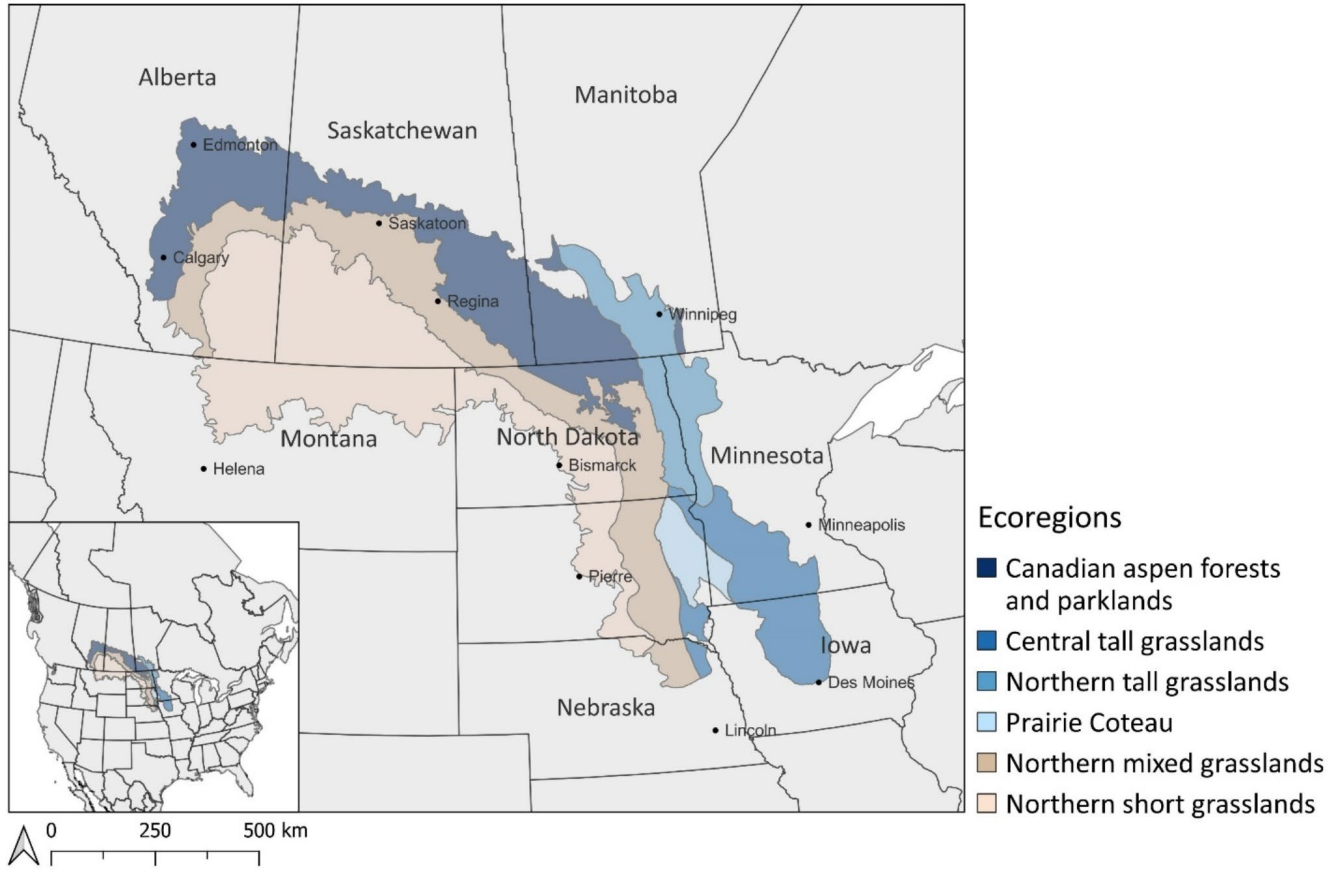
Map of the Prairie Pothole Region with ecoregions that reflect distinct climate and vegetation zones. Adapted from Johnson et al. (2005).

In North America, the Prairie Pothole Region extends through the Canadian Prairies and the Upper Midwest United States (Fig. 1). This area is characterized by palustrine, depressional wetlands, often referred to as prairie potholes or sloughs, that were formed during the Wisconsinan glacial retreat (Klassen 1989; Pennock et al. 2010). These prairie pothole wetlands have been a focus of ecosystem service research, especially in regard to migrating waterfowl habitat, invertebrate biodiversity, water storage regulation, and nutrient cycling (Pennock et al. 2010; Johnson 2019; Elliott et al. 2020; McLean et al. 2021, 2022; Bansal et al. 2023b; Hargiss et al. 2023; Whitfield et al. 2024; Daniel and Rooney 2024). Studies have also investigated the carbon storage potential of prairie pothole wetlands and their overall soil carbon storage, yet there is considerable variability in the reported findings. The variability in wetland soil carbon stock data likely reflects the diverse environmental conditions and management practices across the region, highlighting the need for further synthesis to fully understand the influence of these factors.

Prairie pothole wetlands are often described as being hydrologically isolated, except during wet periods (Hayashi et al. 2016). The wetland pond permanence considers the duration of surface water in the depression and is dependent on the catchment size and its landscape position (Hayashi et al. 2016). The wetland water balance inputs (spring snowmelt, surface runoff, and groundwater discharge) and outputs (evapotranspiration and groundwater recharge) also differ among the prairie pothole wetlands and influence pond permanence (Brannen et al. 2015). The climate is a key factor in these hydrological characteristics, with both inter- and intra- annual climate cycles that are associated with drought and deluge (Johnson et al. 2004; van der Valk and Mushet 2016). Across the Prairie Pothole Region there are distinct climatic zones from arid to semi-humid with a temperature gradient that increases north to south and a precipitation gradient that increases from west to east (Johnson et al. 2005; Millett et al. 2009). These climate zones were used to establish 6 ecoregions for the Prairie Pothole Region that reflect differences in climate, hydrology, and vegetation (Johnson et al. 2005) (Fig. 1). These ecoregions were adapted from Omernik (1995) type III ecoregions in the United States and the soil zones in the Canadian Prairies that reflect the distribution of Chernozemic great groups in the Canadian System of Soil Classification (Pennock et al. 2011). The Brown soil zone aligns with Northern short grasslands ecoregion, Dark Brown soil zone with the Northern mixed grasslands, and the Black soil zones with the Canadian aspen forests and parklands ecoregion.

In the Canadian extent of the Prairie Pothole Region, approximately 70% of the total area is associated with crop production and pasture with a small proportion of native, unmanaged grassland (Wolfe et al. 2019). Similar trends have been observed in the United States as crop production is the major land use and many of the wetlands have been cultivated (Wright and Wimberly 2013). With extensive cultivation, wetland drainage is a common practice to increase the available land and efficiency of production. (Gleason et al. 2008; Bansal et al. 2021).

### Wetland Classification

There are two wetland classification systems, put forward by Stewart and Kantrud (1971) and Millar (1976), that are relevant to the Prairie Pothole Region and attempt to capture some of the wetland variability. While there are additional wetland classification systems and adaptions used in North America, they are often too broad for applications related to wetland management or are developed for specific provincial uses (Cowardin et al. 1979; Warner et al. 1997; Federal Geographic Data Committee 2013; Alberta Environment and Sustainable Resource Development (ESRD) 2015). The Stewart and Kantrud (1971) and Millar (1976) classification systems are both based on pond permanence, vegetation patterns, and surface water salinity. The main difference between the systems is that Millar (1976) also considers wetland size, basin depth, watershed position, and anthropogenic impacts. The use of pond permanence and vegetation patterns for wetland classification can be challenging as these features are dynamic and change over time. Vegetation can also be a difficult identification parameter in cultivated prairie pothole wetlands that are often disturbed and native wetland vegetation is no longer present.

While Millar (1976) incorporates greater detail of the biotic and abiotic wetland features, the broader classes in the Stewart and Kantrud (1971) system can be advantageous when a more generalized series of wetland classes is required. The Stewart and Kantrud (1971) system is comprised of 5 wetland classes that are named in accordance with pond permanence (Class 1– ephemeral, Class 2– temporary, Class 3– seasonal, Class 4– semi-permanent, and Class 5– permanent). In the Prairie Pothole Region, there are greater numbers of ephemeral and temporary wetlands, while seasonal and semi-permanent wetlands cover the greatest area due to their larger size (Stewart and Kantrud 1971).

### Soil Carbon Stocks for Prairie Pothole Wetlands

Through the standardized approach by the Intergovernmental Panel on Climate Change (IPCC), estimates of the amount of soil organic carbon stored in wetlands are calculated using a reference stock which is the amount of carbon stored in the soil based on general climate properties and whether the soil is mineral or organic (IPCC 2006). These reference stocks can be based on values from the IPCC or regional studies. The current IPCC wetland soil organic carbon stocks that are relevant to the Prairie Pothole Region are broadly categorized into “Cool, temperate wet” and “Cool, temperate dry” classes. These classes only consider broad climate variables and are not precise enough to accurately represent variation in climate regimes, wetland classes, or soil characteristics found in the Prairie Pothole Region. The use of reference stocks that are country- or region-specific can improve reference soil organic carbon stocks because they incorporate climate and soil variables that are relevant to the area of interest (IPCC 2006). Field studies that consider these variables are required to generate the data to establish the regional baselines.

### Challenges in Estimating Prairie Pothole Soil Carbon Stocks

Prairie pothole wetlands are capable of storing more soil organic carbon than adjacent crop or grassland areas because they have high vegetation productivity and anaerobic conditions that slow the rate of organic matter decomposition (Badiou et al. 2011). Studies have measured soil carbon in prairie pothole wetlands yet the soil carbon storage estimates are often generalized as being similar across the region. Due to this generalization, there is uncertainty in how factors like climate regime and wetland class influence the amount of soil organic carbon stored in the wetlands. An understanding of wetland carbon storage variation can support decision making on which wetlands in the landscape should be conservation priorities. Understanding these sources of variation is crucial for developing accurate models of carbon storage and informing conservation practices. Furthermore, previous studies on prairie pothole wetlands have addressed field-scale or management induced changes in soil carbon, and there is opportunity to apply their findings to improve our understanding of the landscape-level wetland soil carbon storage.

Another critical aspect that requires attention is the stability of the stored carbon, particularly in terms of its form and depth. Carbon stored in dissolved-, particulate-, or mineral-associated organic matter forms have varying levels of stability and resistance to decomposition. Investigating these forms will provide a clearer picture of how carbon storage in wetlands can be maintained over the long term. The depth at which carbon is stored can influence its susceptibility to environmental changes because it is considered to be more stable at greater depths where it is less vulnerable to microbial decomposition (Harrison et al. 2011). Depth is a particularly important consideration in wetland soils because significant soil carbon deposits can be found below 30 cm (Nahlik and Fennessy 2016). While 0–30 cm is the IPCC (2019) standard soil depth for soil carbon estimates, it can considerably underestimate the wetland soil carbon storage (Harrison et al. 2011). Soil carbon generally decreases with depth in mineral soils, but 27–77% of soil carbon can be stored below 20 cm (Harrison et al. 2011). Additionally, in the Prairie Pothole Region buried surface horizons rich in organic matter can occur below 30 cm in depressions because of historical tillage erosion (VandenBygaart et al. 2012; Konschuh 2013). Inconsistent data management and analysis across studies also poses a significant challenge. Most– but not all– studies have estimated soil organic carbon stocks at depths up to 30 cm on an equivalent soil mass basis to account for differences in soil bulk density. Results are often reported in different units, such as mass-per-area basis (i.e. Mg ha^− 1^) or as a percent (with or without bulk density data) for each depth increment, making it difficult to compare findings and synthesize data across studies (Bansal et al. 2023a).

The objective of this meta-analysis was to assess how wetland soil organic carbon stocks differ across the Prairie Pothole Region by summarizing the existing research. We identified the key factors that explain variability in soil carbon stocks for prairie pothole wetlands then determined average soil organic carbon stock values across ecoregions and land uses. The synthesis of this data from published studies facilitates region-specific soil organic carbon stock estimates for prairie potholes that will more accurately support wetland conservation and soil carbon management programs.

### Methods

A literature review search was conducted using Google Scholar, Scopus, and Web of Science with combinations of the keywords “Prairie Pothole Region”, “prairie pothole”, “prairie”, “wetland”, “depression”, “soil carbon storage”, “soil carbon”, and “soil organic carbon”. The search yielded approximately 134 peer-reviewed papers, government reports, and published datasets. These results were narrowed down to 10 sources from across the Prairie Pothole Region that reported soil organic carbon to at least a depth of 0–30 cm on a mass-per-area basis (i.e. Mg ha^− 1^) or provided soil organic carbon concentration (%) and bulk density data that could be used for unit conversion (Table 1). Some sources shared their raw data for individual wetlands and/or specific landscape positions, but because this was not widely available for all 10 of the sources we relied solely on the average wetland soil organic carbon stocks that were reported in the results section of each source. These sources produced a total of 54 wetland soil organic carbon stock values that we used for further analysis (Table S.1).

**Table 1:** Summary of studies that reported wetland soil organic carbon stocks for 0–30 cm in the Prairie Pothole Region

Ecoregion^1^	Land use	Management practice(s)	Cultivation history	Wetland class^2^	SOC analysis method^3^	*N* ^4^	Source
combined (CAFP, NSG)	grassland	restored, unmanaged	past, none	combined (S, SP, P)	DC840	59	Badiou et al. (2011)
NMG	cropland, grassland	cultivated, rehabilitation, unmanaged	active, past, none	E	DC840	26	Bedard-Haughn et al. (2006)
CAFP	cropland	cultivated, drained	active	combined (E, T, S)	DC1100	42	Brown et al. (2017)
CAFP, NMG	cropland	drained	active	E, T, S	DC1100/RDC400	33	Chizen et al. (2024)
combined (CAFP, CTG, NMG, NTG, PC, NSG)	cropland, grassland	cultivated, drained, rehabilitation, unmanaged	active, past, none	combined (S, SP)	DCICVM	174	Euliss et al. (2006)
combined (NSG, NMG)	grassland	grazed, hayed, rehabilitation, unmanaged	past, none	S	DCICVM	12	Finocchiaro et al. (2014)
NMG	cropland, grassland	cultivated, rehabilitation	active, past	S	DCICVM	16	Gleason et al. (2009)
NSG	cropland, grassland	cultivated, rehabilitation	active, past	–^5^	DCICVM	34	Phillips et al. (2015)
CTG, NMG, NTG	cropland, grassland	cultivated, drained, rehabilitation, restored, unmanaged	active, past, none	S, SP	DCICVM	119	Tangen et al. (2015)
PC	grassland	restored, unmanaged	past, none	combined (T, SP)	DCICVM	6	Zilverberg et al. (2018)

^1^CAFP = Canadian aspen forests and parklands; CTG = Central tall grasslands; NTG = Northern tall grasslands; PC = Prairie Coteau; NMG = Northern mixed grasslands; NSG = Northern short grasslands; combined = multiple ecoregions with specific ones specified in brackets

^2^E = ephemeral; T = temporary; S = seasonal; SP = semi-permanent; P = permanent; combined = multiple wetland classes with specific ones specified in brackets

^3^DC840 = dry combustion at 840 °C; DC1100 = dry combustion at 1100 °C; RDC400 = ramped, dry combustion at 400 °C; DCICVM = total carbon dry combustion with inorganic carbon determined through the volumetric method

^4^N = number of wetlands for calculating the average soil organic carbon stock

^5^A dashed line (–) indicates no available data

For each source, we obtained data on the study location, Johnson et al. (2005) adapted ecoregion(s), Stewart and Kantrud (1971) wetland classification(s), land use(s), management practice(s), cultivation history, sampling position(s) within the wetland, sampling depth increment, average soil organic carbon stock, soil organic carbon analysis method(s), and number of wetlands used to calculate their average soil organic carbon stock. The types of wetland management described in the literature were summarized and grouped to their specific land uses and management categories (Table 2). We identified 10 management practices utilized between the cropland and grassland land uses. For the restoration management category, the reversal of drainage was required and wetlands with any duration since restoration were included. Rehabilitation management was assigned to wetlands that had not been previously drained but native vegetation was re-established following cultivation or forage production (IPCC 2014). Cultivation history was also determined based on the source site descriptions that indicated whether the wetland had any history of active or previous crop production.

**Table 2:** Summary of wetland management practices for crop production and grassland land uses in the Prairie Pothole Region

Land use	Management	Description
cropland	cultivated	active or recent crop production with no history of drainage
	drained	wetland has been hydrologically altered through drainage, and has active or recent use for crop production
	rehabilitation	native vegetation has been restored and has no history of drainage, but was cultivated in the past
	restored^1^	wetland had historically been drained and cultivated, but the hydrology and native vegetation has been restored
	field edge^1^	adjacent to crop field, with native vegetation and no history of drainage or cultivation
grassland	grazed	active grazing management with no history of drainage
	hayed	regularly hayed with no history of drainage
	rehabilitation	native vegetation has been restored, has no history of drainage, and was managed for agricultural use in the past
	restored	wetland had historically been drained and was managed for agricultural use in the past, but the hydrology and native vegetation has been restored
	unmanaged	native vegetation and not actively managed for agricultural use

^1^These management categories were not considered in the 10 studies included in this meta-analysis but were reconized as important

Data mining approaches were used to identify factors that explained variability in the soil carbon dataset. Studies that averaged soil organic carbon across multiple ecoregions or wetland classes were excluded from these analyses. All analyses were conducted in R (R Core Team 2023). The soil organic carbon analysis method was also not included because it exhibited dependence with several of the other variables while the model assumptions were evaluated. The land management factor represented the overall land use (grassland or cropland) as well as the management practices (i.e., undrained cultivated, drained cultivated, restored pasture, etc.) (Table 2). The variable importance for soil carbon storage was assessed using multiple linear regression model and the Lindeman, Merenda, and Gold (LMG) relative importance with the caret and relaimpo packages, respectively (Lindeman et al. 1980; Grömping 2006; Kuhn 2008). To evaluate the usefulness of considering land management for wetland soil carbon storage, we compared the importance of the variables in a simplified model with ecoregion, land use, and wetland class to a more comprehensive model with ecoregion, land management, and wetland class.

Average soil organic carbon stocks were determined for each ecoregion and land use combination. We were unable to calculate the soil organic carbon stocks for the ecoregion × wetland class factor levels due to a lack of representation of the wetland classes (i.e., temporary or semi-permanent) in each of the ecoregions. A multiple linear regression was used to compare the average soil organic carbon stock values among ecoregions and land uses. A logarithmic transformation was used to ensure that the model assumptions of equal variance and normality were met. When ecoregion or land use were significant factors in the model, post-hoc analysis with TukeyHSD was used to evaluate significant differences between the factor levels through the emmeans and multcomp packages (Hothorn et al. 2008; Lenth 2023).

## Results

The overall wetland soil organic carbon stock across the Prairie Pothole Region was estimated as 124.5 Mg C ha^− 1^ for 0–30 cm based on data from the literature. However, specific factors were identified as having a considerable effect on these carbon stocks and should be used as categories for developing more specific estimates of wetland soil carbon stocks. Wetland class accounted for the greatest amount of variability in soil organic carbon stocks and was ranked as having the highest relative importance in both the simplified model and the comprehensive model (Table 3). The dataset compiled from existing research included observations for ephemeral, temporary, seasonal, and semi-permanent wetlands. While there were some studies that included research on permanent wetlands, they had combined the average soil organic carbon stocks with values from other wetland classes and thus they were removed from the variable importance analysis.

**Table 3:** Prairie pothole wetland factors that account for variability in soil organic carbon (SOC) stocks. The simplified model uses land use (grassland or cropland) while the comprehensive model uses more specific land management practices (i.e., restored pasture, drained cultivated, etc.). Factors were ranked based on the variability explained (VE) from the ANOVA output and the relative importance (RI) from the Lindeman, Merenda, and Gold (LMG) method

Simplified Model *R*^2^ = 17%					Comprehensive Model *R*^2^ = 47%			
Factor	Rank	SOC VE (%)	RI (%)		Factor	Rank	SOC VE (%)	RI (%)
wetland class	1	11.2	67.1		wetland class	1	26.6	40.6
ecoregion	2	6.0	32.4		land management	2	14.4	46.3
land use	3	0.3	0.5		ecoregion	3	6.0	13.1

The ecoregion explained 6% of the soil carbon stock variability in the regression model (Table 3). We found that the soil organic carbon stocks differed significantly across ecoregions, being highest in the Central tall grasslands and Northern tall grasslands, and lowest in the Northern short grasslands (Table 4). The Canadian aspen forests and parkland, Prairie Coteau, and Northern short grasslands followed this trend with precipitation and temperature gradients but were significantly different from one another. This was likely due to the high standard error and low sample numbers in these ecoregions.

**Table 4:** Average soil organic carbon (SOC) stocks from 0–30 cm in prairie pothole wetlands for each ecoregion was summarized from 11 studies and further cateogrized into grassland and cropland land uses. Standard error is provided in brackets

Ecoregion^1^	Cropland			Grassland			Combined Land Use	
	*N* ^2^	SOC (Mg C ha^− 1^)		**N**	SOC (Mg C ha^− 1^)		**N**	SOC (Mg C ha^− 1^)
CTG	2	145.9 (9.52)		4	127.8 (17.10)		6	133.9 (11.72) a^3^
NTG	4	126.7 (3.17)		6	135.9 (5.62)		10	132.3 (3.76) a
Combined	2	97.7 (1.40)		9	135.1 (11.75)		11	128.3 (10.55) a
CAFP	5	126.4 (33.48)		–	–		5	126.4 (33.48) ab
NMG	10	123.0 (11.25)		8	126.8 (7.93)		18	124.7 (6.99) a
PC	–^5^	–		2	96.5 (15.50)		2	96.5 (15.50) ab
NSG	1	54.9 (*NA*)^4^		1	61.1 (*NA*)		2	58.0 (3.14) b

^1^CAFP = Canadian aspen forests and parklands; CTG = Central tall grasslands; NMG = Northern mixed grasslands; NSG = Northern short grasslands; NTG = Northern tall grasslands; PC = Prairie Coteau; Combined = two or more ecoregions reported together

^2^N = number of wetlands included for calculating the average soil organic carbon stock

^3^Differences between ecoregions are denoted lowercase letters (α = 0.05)

^4^Averages with only one site have an *NA* for standard error since it could not be determined

^5^A dashed line (–) indicates no available data

Land use (cropland or grassland) on its own was not a significant factor in soil organic carbon storage as it only explained 0.3% of the variability in the dataset and had a low relative importance from the LMG metric (Table 3). However, when the land use was replaced with more specific land management factor levels comprehensive model, it was identified as the second most important variable in explaining soil organic carbon variability (Table 3). Due to limited representation of the land management practices across the ecoregions, we were unable to calculate soil carbon stock values for this factor. Soil carbon stock values with land use showed no significant effect without the specific land management practices (Table 4).

There were 4 general methods used to measure soil organic carbon and they all involved the dry combustion approaches. In the United States, the dominant analytical method for soil organic carbon was the subtraction of soil inorganic carbon using the volumetric method from total soil carbon measured from dry combustion. In Canada, soil organic carbon was measured by dry combustion at 840 °C, pre-treatment of soil with sulfuric acid to remove inorganic carbon then dry combustion at 1100 °C, or ramped dry combustion with organic carbon specifically measured at 400 °C. All the studies collected soil samples to depth of at least 30 cm. They used a variation of transect sampling or transect and grid sampling, with the exception of Phillips et al. (2015), which used plot sampling within the wetland depressions.

## Discussion

### Key Drivers of Soil Carbon Variability

Climate factors, including temperature and precipitation, are recognized as important controls on soil organic carbon accumulation across land uses. The climate trends reflected in the ecoregions for the Prairie Pothole Region were associated with lower soil carbon stocks in warm, dry areas (i.e. Northern short grasslands) and higher soil carbon stocks in cool, humid areas (i.e. Northern tall grasslands and Central tall grasslands). However, we were unable to clearly differentiate among the ecoregions that have climates that are more similar to one another (i.e. Northern mixed grasslands, Central tall grasslands, Northern tall grasslands, Canadian aspen forests and parkland). In the Canadian Prairies, several research projects have observed differences between with soil organic carbon being lowest in the Northern short grasslands and increased in the Northern mixed grasslands, then highest in the Canadian aspen forests and parkland (Janzen et al. 1998; Sorenson et al. 2021). The main reason that we did not observe these clear contrasts between the ecoregions is because of the limited number of prairie pothole wetland studies that measured soil organic carbon and met our data criteria. This is also constrained our interpretation as there was an uneven distribution of studies across the ecoregions and land uses. For instance, there was only one study that provided specific soil carbon values within the Northern short grasslands ecoregion. Considering that this ecoregion has the third highest number of wetlands per square kilometer in the Prairie Pothole Region, more soil carbon data is required to ensure that it is accurately represented (Millett et al. 2009). Additionally, two of the 10 studies reported their wetland soil organic carbon stocks across multiple ecoregions. Based on all of the data in summarized in this study, we calculated that prairie pothole wetlands had an overall average soil organic carbon stock of 124.5 Mg C ha^− 1^ which was relatively similar to the IPCC (2014) estimate of 128 Mg C ha^− 1^ for mineral wetland soils in temperate-moist climates.

The variability in soil carbon storage was largely explained by wetland class. This is expected as greater vegetation productivity and longer saturated conditions supports the accumulation of organic matter. Greater soil organic carbon stocks have been observed for wetland classes that have a longer pond permanence (Bansal et al. 2021; Chizen et al. 2024). Tangen et al. (2015) evaluated soil carbon storage in seasonal and semi-permanent wetlands for various land uses, but there were no distinct trends with wetland class. In our meta-analysis, we found that wetland class was not often explicitly included in explaining soil organic carbon stocks. Bansal et al. (2021) acknowledged the differences in soil organic carbon stocks with wetland class in their meta-analysis but focused on the response of soil carbon to land management by averaging the soil carbon stock values across all of the wetland classes. The ability to accurately calculate average soil carbon stocks for each wetland class would require more detailed data from each of these classes across ecoregions and land management.

The wetland soil carbon storage capacity is an equilibrium of net carbon additions and losses that is achieved over decades, and this can be disrupted by changes in land use or management (Janzen et al. 1998). In response to these changes, the soil organic carbon stock values that we measure or estimate will approach a new equilibrium over time (Janzen et al. 1998). Climate fluctuations can also impact the soil carbon equilibrium as shifts in precipitation and temperature can influence plant productivity and rates of organic matter decomposition (Yu et al. 2021). The temporal factor associated with management practices has been investigated by Badiou et al. (2011) and found that long-term restored wetlands (> 5 years) had greater carbon storage than short-term restored wetlands (< 5 years). However, there is an absence of data for restored wetlands with shorter pond permanence that are more frequently subject to drainage. A study found no significant differences in soil organic carbon stocks from 0–15 cm among undrained, cultivated wetlands and cultivated wetlands that had been drained less than 34 years ago (Brown et al. 2017). The wetlands that have been drained and cultivated for over 34 years had more similar soil organic carbon values to the mid-slope landscape position that was outside of the wetland boundary (Brown et al. 2017). The concentrations at the 15–30 or 30–60 cm depths were similar across the time periods since drainage installation (Brown et al. 2017). Euliss et al. (2006) estimated that wetland drainage and subsequent cultivation results in an average loss of 10.1 Mg C ha^− 1^ in the upper 15 cm of the soil profile, but this was not statistically different between drained and undrained cultivated wetlands. The drained and undrained cultivated wetlands likely had similar soil organic carbon stocks because both have a cultivation history, which is known to have a major impact on soil organic carbon storage (Pennock et al. 1994; Euliss et al. 2006; Zilverberg et al. 2018). The combination of drainage and cultivation likely improved water infiltration and buried original soil horizons via tillage erosion, allowing for soil organic carbon storage at greater depths (Pennock et al. 1994; Bedard-Haughn et al. 2006; Brown et al. 2017; Zarrinabadi et al. 2023). While these studies have provided valuable insights into how management influence wetland soil organic carbon, there is a need for further research across the Prairie Pothole Region to more accurately estimate the changes in soil organic carbon, especially for cultivated land uses that have not been drained.

Tangen and Bansal (2020) recently estimated prairie pothole wetland soil carbon storage through the analysis of a comprehensive dataset from the United States that included several of the same studies as this analysis. They broadly grouped the wetlands into natural, restored, and cropped, then estimated carbon for various landscape positions. Their research provided improved carbon storage estimates by considering these environmental and management factors. To advance wetland carbon storage estimates and predictive modelling, further research should incorporate studies from both the United States and Canada, as well as a wider range of management practices.

### Methods for Measuring Wetland Soil Carbon Storage

Standardized data collection and reporting methods would help create a more cohesive and comprehensive understanding of wetland carbon storage in the Prairie Pothole Region. Many studies measure soil organic carbon, but do not provide values on a mass-per-area basis for standard depth increments (i.e., 0–30 cm or 0–100 cm) which limits the usability of the data for estimating wetland soil carbon storage. Providing soil organic carbon measures on a mass-per-area basis or including soil parameters to calculate this can support future landscape modelling of soil organic carbon.

A constraint of this research is that we did not consider whether studies calculated soil organic carbon on a fixed mass or equivalent soil mass basis. While most studies reported how they calculated the soil organic carbon, there were some that did not so we were unable to consider this variable in our analysis. The equivalent soil mass method accounts for differences in carbon density within the soil profile, and is especially important to consider when comparing across land uses or management practices where differences in soil bulk density are expected (Ellert and Bettany 1995; Wendt and Hauser 2013; Bansal et al. 2023a). With these soil organic carbon values, it is also important to consider how they are scaled up to the individual wetland area. The method of using soil carbon values at specific landscape positions to proportionally calculate wetland soil organic carbon storage can more accurately represent the within-wetland soil carbon variability (Tangen and Bansal 2020); however, delineating wetland landscape positions can be time and labour intensive. Further advancements can be made with remote sensing and geographic information system (GIS) technology to facilitate this process.

Another aspect that should be considered while measuring wetland soil carbon is sampling depth. The majority of research reports soil organic carbon storage for 0–30 cm. However, shallow sampling depths may miss significant amounts of carbon stored deeper in the soil profile, leading to underestimates of the total soil organic carbon storage potential. To more adequately understand processes of soil organic carbon storage and the effects of land use change, research suggests considering soil organic carbon storage to greater soil depths, ideally 1 m, and including soil organic carbon measurements from organic horizons (Olson and Al-Kaisi 2015; FAO 2020).

### Wetland Classification Systems

In the Prairie Pothole Region, wetland classification is vital for understanding and managing soil carbon storage and other ecosystem services. We identified that the wetland classes from Stewart and Kantrud (1971) were important in explaining variability in soil organic carbon. A study by van der Kamp et al. (2016) recognized inconsistencies in the use of wetland classes based on hydrology and vegetation characteristics because they are dynamic and expected to evolve over time, especially with climate change. There is a need to consider how we can further develop the wetland classification system to ensure that it is robust and relevant for land management decision-making.

The inclusion of hydric soil features in grouping wetlands is one strategy to support evidence of periodic or prolonged saturated soil conditions. Wetland mineral soils are defined by the IPCC (2006) as having an aquic (Soil Taxonomy) or Gleysolic soil classification (World Reference Base, Canadian System of Soil Classification). This strategy of relying on soil order is a starting point, but it overlooks some ephemeral or temporary prairie pothole wetlands that may not have saturated conditions for long enough to develop dominant gleyed features. Stewart and Kantrud (1971) as well as other relevant wetland classification systems for the Prairie Pothole Region do not use hydric soil features in assigning wetland class. There is opportunity to leverage the identification of hydric soil features in addition to landscape topography to support the existing wetland classification system. By refining classification systems to better reflect these diverse factors, policymakers and researchers can establish conservation strategies that maximize the ecological and carbon storage benefits of prairie pothole wetlands.

## Conclusions and Recommendations

Our analysis highlights the significant influence of climate regimes, land use practices, wetland management strategies, and hydrological conditions on soil carbon estimates. Understanding these variables is crucial for accurate soil carbon reporting, particularly in diverse wetland ecosystems such as the prairie pothole wetlands across Canada and the United States. Utilizing existing data to model soil carbon storage in these regions provides valuable insights into the spatial variability and amount of soil carbon stored in these wetlands. Structured soil carbon surveys and advancements in monitoring technologies are essential for capturing the dynamics of soil carbon storage at the landscape level. These efforts enable more precise and reliable estimates, which are fundamental for informing greenhouse gas modelling and policy development.

Based on our findings, we propose the following recommendations to advance wetland soil carbon research for the Prairie Pothole Region:

1. Modelling Wetland- and Landscape-Level Soil Carbon Storage

The development of predictive models for wetland soil carbon across the Prairie Pothole Region will help assess how changes in agricultural practices or wetland management affect soil carbon storage over years or decades. These models can guide land managers and policymakers in adopting practices that enhance carbon storage and mitigate greenhouse gas emissions. Future studies should explicitly account for climate regimes, land management, topography, hydrological conditions, and other relevant variables to enhance the accuracy of soil carbon estimates. Integrating these comprehensive datasets will refine predictions and support targeted conservation efforts.

2. Soil Carbon Measurement and Monitoring Technologies

Harmonizing soil carbon data collection methods and innovation in advanced soil carbon monitoring tools will enable large-scale assessments of soil carbon storage for the Prairie Pothole Region. Publicly available remote sensing sources, proximal soil sensors, and soil carbon models are examples of technology advancements that will especially support enhancing wetland soil carbon monitoring. However, prior to the adoption of new technology we need to thoroughly validate the accuracy and consider suitability, cost, and efficiency, relative to existing methods. Improving our capacity for estimating soil organic carbon will also support monitoring, reporting, and verification protocols for carbon credit programs. Greater accessibility of these tools can enhance the ability for landowners to benefit from financial incentives for sustainable land management.

3. Wetland Classification for Meaningful Ecosystem Service Assessment

Adjusting wetland classification systems to better capture the diversity and specific characteristics of different wetland types will improve the accuracy of soil carbon estimates. Wetlands are of interest to various disciplines because of their diverse ecological functions, resource potential, and dynamic biogeochemical processes. The development of a revised wetland classification system for prairie pothole wetlands will require interdisciplinary conversations to ensure that it is functional and relevant for each of these groups. An improved classification system will aid in more precise modeling and reporting of soil carbon, facilitate better assessments of ecosystem services, and support informed conservation and management decisions.

## Electronic Supplementary Material

Below is the link to the electronic supplementary material.

Supplementary Material 1

## Data Availability

A summary of the data used in this study is provided in the supplementary material.
