# Supplementary material for: Drivers of Soil Carbon Variability in North America’s Prairie Pothole Wetlands: A Review
Source: Wetlands (Wilmington). 2025 Jan 30;45(1):18. doi: 10.1007/s13157-025-01898-9 (PMC11782295; doi:10.1007/s13157-025-01898-9)
Supplement: Supplementary file 1 — Supplementary Material 1 [file 13157_2025_1898_MOESM1_ESM.docx]

**Drivers of soil carbon variability in North America’s**

**prairie pothole wetlands: A review**

Chantel Chizen^a^, Angela Bedard-Haughn^ab^

^a^ Department of Soil Science, College of Agriculture and Bioresources, University of Saskatchewan, Saskatoon, Saskatchewan, Canada

^b^ Global Institute for Water Security, University of Saskatchewan, Saskatoon, SK, Canada

**Corresponding Author:** Chantel Chizen (chantel.chizen@usask.ca)

ORCID iDs

Chantel Chizen: 0000-0001-7680-8610

Angela Bedard-Haughn: 0000-0002-3971-8509

# Supplementary Material

**Table S.1.** Detailed summary of studies used to assess factors that account for variability in soil organic carbon (SOC) stocks for prairie pothole wetlands.

| **Location**^1^ | **Ecoregion**^2^ | **Land-use** | **Management practice(s)** | **Cultivation history** | **Wetland class**^3^ | **SOC analysis method**^4^ | **SOC**  **(Mg ha^-1^)** | **N**^5^ | **Source** |
| --- | --- | --- | --- | --- | --- | --- | --- | --- | --- |
| AB, SK, MB | combined  (CAFP, NSG) | grassland | restored | past | combined  (S, SP, P) | DC840 | 121.00 | 14 | Badiou et al. (2011) |
| AB, SK, MB | combined  (CAFP, NSG) | grassland | restored | past | combined  (S, SP, P) | DC840 | 165.00 | 26 | Badiou et al. (2011) |
| AB, SK, MB | combined  (CAFP, NSG) | grassland | unmanaged | none | combined  (S, SP, P) | DC840 | 205.00 | 19 | Badiou et al. (2011) |
| SK | NMG | cropland | cultivated | active | E | DC840 | 87.20 | 7 | Bedard-Haughn et al. (2006) |
| SK | NMG | cropland | rehabilitation | past | E | DC840 | 168.60 | 7 | Bedard-Haughn et al. (2006) |
| SK | NMG | grassland | unmanaged | none | E | DC840 | 175.10 | 12 | Bedard-Haughn et al. (2006) |
| SK | CAFP | cropland | cultivated | active | combined  (E, T, S) | DC1100 | 78.60 | 10 | Brown et al. (2017) |
| SK | CAFP | cropland | drained | active | combined  (E, T, S) | DC1100 | 65.80 | 32 | Brown et al. (2017) |
| SK | CAFP | cropland | drained | active | E | DC1100/RDC400 | 83.00 | 2 | Chizen et al. (2024) |
| SK | CAFP | cropland | drained | active | S | DC1100/RDC400 | 241.30 | 5 | Chizen et al. (2024) |
| SK | CAFP | cropland | drained | active | T | DC1100/RDC400 | 163.40 | 1 | Chizen et al. (2024) |
| SK | NMG | cropland | drained | active | E | DC1100/RDC400 | 54.50 | 7 | Chizen et al. (2024) |
| SK | NMG | cropland | drained | active | S | DC1100/RDC400 | 160.40 | 5 | Chizen et al. (2024) |
| SK | NMG | cropland | drained | active | T | DC1100/RDC400 | 126.50 | 13 | Chizen et al. (2024) |
| MT, ND, SD, MN, IA | combined  (CAFP, CTG, NMG, NTG, PC, NSG) | cropland | drained | active | combined  (S, SP) | DCICVM | 99.10 | 31 | Euliss et al. (2006) |
| MT, ND, SD, MN, IA | combined  (CAFP, CTG, NMG, NTG, PC, NSG) | cropland | cultivated | active | combined  (S, SP) | DCICVM | 96.30 | 38 | Euliss et al. (2006) |
| MT, ND, SD, MN, IA | combined  (CAFP, CTG, NMG, NTG, PC, NSG) | grassland | unmanaged | none | combined  (S, SP) | DCICVM | 106.10 | 40 | Euliss et al. (2006) |
| MT, ND, SD, MN, IA | combined  (CAFP, CTG, NMG, NTG, PC, NSG) | grassland | rehabilitation | past | combined  (S, SP) | DCICVM | 96.50 | 65 | Euliss et al. (2006) |
| SD | combined  (NSG, NMG) | grassland | unmanaged | none | S | DCICVM | 112.70 | 3 | Finocchiaro et al. (2014) |
| SD | combined  (NSG, NMG) | grassland | grazed | none | S | DCICVM | 163.80 | 3 | Finocchiaro et al. (2014) |
| SD | combined  (NSG, NMG) | grassland | rehabilitation | past | S | DCICVM | 125.10 | 3 | Finocchiaro et al. (2014) |
| SD | combined  (NSG, NMG) | grassland | hayed | past | S | DCICVM | 120.50 | 3 | Finocchiaro et al. (2014) |
| SD | NMG | cropland | cultivated | active | S | DCICVM | 95.70 | 8 | Gleason et al. (2009) |
| SD | NMG | grassland | rehabilitation | past | S | DCICVM | 104.00 | 8 | Gleason et al. (2009) |
| ND | NSG | cropland | cultivated | active | NA | DCICVM | 54.85 | 17 | Phillips et al. (2015) |
| ND | NSG | grassland | rehabilitation | past | NA | DCICVM | 61.13 | 17 | Phillips et al. (2015) |
| MN, IA | CTG | cropland | drained | active | S | DCICVM | 136.34 | 4 | Tangen et al. (2015) |
| MN, IA | CTG | grassland | unmanaged | none | S | DCICVM | 142.06 | 4 | Tangen et al. (2015) |
| MN, IA | CTG | grassland | restored | past | S | DCICVM | 103.71 | 11 | Tangen et al. (2015) |
| MN, IA | CTG | cropland | drained | active | SP | DCICVM | 155.37 | 4 | Tangen et al. (2015) |
| MN, IA | CTG | grassland | unmanaged | none | SP | DCICVM | 169.38 | 4 | Tangen et al. (2015) |
| MN, IA | CTG | grassland | restored | past | SP | DCICVM | 96.22 | 11 | Tangen et al. (2015) |
| ND | NMG | cropland | cultivated | active | S | DCICVM | 129.73 | 4 | Tangen et al. (2015) |
| ND | NMG | cropland | drained | active | S | DCICVM | 129.97 | 4 | Tangen et al. (2015) |
| ND | NMG | grassland | unmanaged | none | S | DCICVM | 137.02 | 4 | Tangen et al. (2015) |
| ND | NMG | grassland | rehabilitation | past | S | DCICVM | 121.79 | 4 | Tangen et al. (2015) |
| ND | NMG | grassland | restored | past | S | DCICVM | 116.68 | 4 | Tangen et al. (2015) |
| ND | NMG | cropland | cultivated | active | SP | DCICVM | 121.62 | 4 | Tangen et al. (2015) |
| ND | NMG | cropland | drained | active | SP | DCICVM | 155.85 | 4 | Tangen et al. (2015) |
| ND | NMG | grassland | unmanaged | none | SP | DCICVM | 133.35 | 4 | Tangen et al. (2015) |
| ND | NMG | grassland | rehabilitation | past | SP | DCICVM | 115.95 | 4 | Tangen et al. (2015) |
| ND | NMG | grassland | restored | past | SP | DCICVM | 110.30 | 5 | Tangen et al. (2015) |
| MN | NTG | cropland | cultivated | active | S | DCICVM | 120.01 | 4 | Tangen et al. (2015) |
| MN | NTG | cropland | drained | active | S | DCICVM | 134.42 | 4 | Tangen et al. (2015) |
| MN | NTG | grassland | unmanaged | none | S | DCICVM | 133.89 | 4 | Tangen et al. (2015) |
| MN | NTG | grassland | rehabilitation | past | S | DCICVM | 143.00 | 4 | Tangen et al. (2015) |
| MN | NTG | grassland | restored | past | S | DCICVM | 147.87 | 4 | Tangen et al. (2015) |
| ND | NTG | cropland | cultivated | active | SP | DCICVM | 123.43 | 4 | Tangen et al. (2015) |
| ND | NTG | cropland | drained | active | SP | DCICVM | 129.11 | 4 | Tangen et al. (2015) |
| ND | NTG | grassland | unmanaged | none | SP | DCICVM | 121.43 | 4 | Tangen et al. (2015) |
| ND | NTG | grassland | rehabilitation | past | SP | DCICVM | 118.28 | 4 | Tangen et al. (2015) |
| ND | NTG | grassland | restored | past | SP | DCICVM | 151.05 | 4 | Tangen et al. (2015) |
| SD | PC | grassland | unmanaged | none | combined  (T, SP) | DCICVM | 112.00 | 3 | Zilverberg et al. (2018) |
| SD | PC | grassland | restored | past | combined  (T, SP) | DCICVM | 81.00 | 3 | Zilverberg et al. (2018) |

^1^AB = Alberta; SK = Saskatchewan; MB = Manitoba; MT = Montana; ND = North Dakota; SD = South Dakota; MN = Minnesota; IA = Iowa.

^2^CAFP = Canadian aspen forests and parklands; CTG = Central tall grasslands; NMG = Northern mixed grasslands; NSG = Northern short grasslands; NTG = Northern tall grasslands; PC = Prairie Coteau.

^3^E = ephemeral; T = temporary; S = seasonal; SP = semi-permanent; P = permanent.

^4^DC840 = dry combustion at 840°C; DC1100 = dry combustion at 1100°C; RDC400 = ramped, dry combustion at 400°C; DCICVM = total carbon dry combustion with inorganic carbon determined through the volumetric method.

^5^ N = number of wetlands.
